# COST-UTILITY OF A MULTICOMPONENT INTERVENTION FOR FIBROMYALGIA VERSUS USUAL CARE: A PRAGMATIC RANDOMISED CONTROLLED TRIAL

**DOI:** 10.2340/jrm.v55.12361

**Published:** 2023-12-27

**Authors:** Victoria Mailen ARFUCH, Carina AGUILAR MARTÍN, Anna BERENGUERA, Rosa CABALLOL ANGELATS, Alessandra QUEIROGA GONÇALVES, Noèlia CARRASCO-QUEROL, Gemma GONZÁLEZ SERRA, Maria Cinta SANCHO SOL, Immaculada FUSTÉ ANGUERA, Emilie FRIBERG, Emma PETTERSSON, Marc CASAJUANA

**Affiliations:** 1 Terres de l'Ebre Research Support Unit, Jordi Gol I Gurina Primary Health Research Institute Foundation (IDIAPJGol), Tortosa; 2 Department of Pediatrics, Obstetrics and Gynecology, and Preventive Medicine and Public Health, Autonomous University of Barcelona, Bellaterra, Spain; 3 Division of Insurance Medicine, Department of Clinical Neuroscience, Karolinska Institutet, Stockholm, Sweden; 4 UEvaluation Unit, Directorate of Primary Care Terres de l'Ebre, Territorial Management of Terres de l'Ebre, Catalan Institute of Health (ICS), Tortosa; 5 Central Research Unit, Jordi Gol I Gurina Primary Health Research Institute Foundation (IDIAPJGol), Barcelona; 6 Department of Nursing, University of Girona, Plaça de Sant Domènec, Girona; 7 Primary Care Center (CAP) El Temple, Territorial Management of Terres de l’Ebre, Catalan Health Institute (ICS), Tortosa; 8 Unit of Expertise in Central Sensitization Syndromes Terres de l'Ebre, Territorial Management of Terres de l'Ebre, Catalan Institute of Health (ICS), Tortosa; 9 Family and Community Medicine Teaching Unit Tortosa-Terres de L’Ebre, Catalan Institute of Health (ICS), Tortosa; 10 Rehabilitation and Physical Medicine Service, Tortosa Verge de la Cinta Hospital, Territorial Management of Terres de l'Ebre, Catalan Health Institute (ICS), Tortosa; 11 Adult Mental Health Center (CSMA) of Fundació Pere Mata Terres de l’Ebre, Tortosa, Spain

**Keywords:** cost-utility analysis, fibromyalgia syndrome, health economic evaluation, multicomponent intervention, primary care

## Abstract

**Objective:**

To perform an economic evaluation on a multicomponent intervention programme for patients with fibromyalgia syndrome compared with usual clinical practice in primary care.

**Design:**

A cost-utility analysis was conducted alongside a pragmatic randomised controlled trial (ClinicalTrials.gov: https://clinicaltrials.gov/ct2/show/record/NCT04049006) from a societal perspective, a human capital approach, and a 1-year time horizon.

**Patients:**

Patients diagnosed with fibromyalgia syndrome from the public health system in south Catalonia, Spain.

**Methods:**

Crude and adjusted incremental cost-utility ratios were estimated to compare the treatment strategies based on cost estimations (direct medical costs and productivity losses) and quality-adjusted life years. One-way and 2-way deterministic sensitivity analyses were performed.

**Results:**

The final analysed sample comprised 297 individuals, 161 in the intervention group and 136 in the control group. A crude incremental cost-utility ratio of € 1,780.75 and an adjusted ratio of € 851.67 were obtained, indicating that the programme significantly improved patients’ quality of life with a cost-increasing outcome that fell below the cost-effectiveness threshold. The sensitivity analysis confirmed these findings when varying large cost components, and showed dominance when increasing session attendance.

**Conclusion:**

The proposed multicomponent intervention programme was cost-effective compared with usual care for fibromyalgia, which supports its addition to standard practice in the regional primary care service.

LAY ABSTRACT

Following the gold standard trial design for evaluating healthcare technologies, a health economic evaluation was conducted on a new multidisciplinary intervention programme for patients with fibromyalgia syndrome in primary care centres in Catalonia, Spain. Estimations of direct healthcare and social costs were assessed jointly with the effect on quality of life, in order to compare the new intervention with usual clinical care. The results showed that the programme proved its cost-utility for society, as patients’ perceived health improvements outweighed the cost increase, according to the maximum price accepted for an extra unit of quality of life in Spain. These findings were verified when cost estimations were corrected with the actual health expenditure. In addition, the intervention implementation scheme demonstrated cost-saving potential by increasing participants’ session attendance to at least 66%. Therefore, patients and society could benefit from this new therapeutic strategy for fibromyalgia if adopted by the regional services portfolio.

Fibromyalgia syndrome (FMS) is one of the most frequent pain disorders among rheumatic illnesses (1), particularly in women (2). Its prevalence has been estimated at between 0.2% and 6.6% globally (3) and 2.45% in the Spanish population (4). In addition, Ursini et al. (5) have shown FMS to be a new facet of the spectrum of post-COVID-19 syndrome, given the similarity of their clinical picture.

FMS is currently classified as a central sensitivity syndrome (6, 7) and is characterized by widespread musculoskeletal pain and fatigue as core clinical diagnostic criteria (8) owing to the lack of biomarkers and specific medical tests. While its aetiopathogenesis remains unclear and controversial (9), FMS entails a high cost for society (10–15), estimated at €12,993 million annually in Spain in 2017 (15). Its chronic and disabling nature significantly impacts patients’ quality of life (QoL) and functionality, leading to high productivity losses (16). However, there is no gold standard treatment for FMS. International guidelines suggest a multidisciplinary approach (17–19) based on promising research experiences, but the evidence is limited (20).

Economic evaluations in health management are gaining importance in decision-making. A recently published systematic review on economic evaluations for non-pharmacological treatment strategies for FMS highlighted the need for similar studies alongside randomized controlled trials (RCTs) (21). Cost-utility analysis (CUA) provides a valuable tool for assessing cost and patients’ QoL and comparing them with other health technologies, in order to inform resource allocation (22). Previous studies on CUA and non-pharmacological treatment approaches for FMS conducted in Spain (23–28) have found favourable results supporting these new strategies from a health-economic standpoint. However, there is scope for further research in this field.

Alongside a pragmatic RCT, this study aims to conduct a health economic evaluation to assess the cost-utility of a novel multicomponent intervention (MCI) programme for patients with FMS compared with usual clinical care (UCC) in primary care settings in south Catalonia, Spain. The results of this study are expected to provide helpful evidence for decision-makers in healthcare management.

## METHODS

### Design

A CUA was conducted alongside a pragmatic RCT (29) (ClinicalTrials.gov: NCT04049006) on the effectiveness of a MCI programme for patients with FMS compared with the UCC. This type of economic evaluation is particularly suitable considering the decremental effect of FMS on patients’ QoL. Likewise, the study adopted a societal perspective (30), which means that all available registered costs incurred by the patient, the healthcare funder, and society were considered. In addition, this study covered a 1-year time horizon in light of the reported large impact of FMS on productivity losses and QoL.

Moreover, the human capital approach (31), which assumes workers value their earnings, was implemented due to data availability in the regional electronic medical record called “Estació Clínica d'Atenció Primària” (eCAP), where only full sick-leave days prescribed by the general practitioner (GP) are recorded. Finally, this study was designed following the Consolidated Standards of Reporting Trials (CONSORT) guidelines for pragmatic trials (32), the Consolidated Health Economic Evaluation Reporting Standards (CHEERS) (33) and the UK Medical Research Council guidance for complex interventions (34).

### Setting

The UCC for FMS in the Spanish National Health Service system consists of cost-free medical services, including diagnosis, treatment and pharmacological guidance with co-payment (35) for medicines (36). Territorial Management of Terres de l'Ebre (GTTE), Catalan Institute of Health (ICS) follows Catalonian medical practice guidelines for treating FMS within the Central Sensitivity Syndromes Specialized Units located in primary care centres and hospitals, where multidisciplinary healthcare has been targeted as the gold standard treatment strategy since 2016 (37).

The proposed MCI was developed according to this framework, and consists, in addition to the UCC, of a total 24-h group programme (2-h week) integrating health education sessions, physical activity and cognitive behavioural therapy (CBT) delivered by a nurse and a GP, a physiotherapist and psychologist, respectively. Content details of the programme can be found in the study protocol (29). This intervention aimed to strengthen routine practice by providing non-pharmacological strategies for symptomatic control to improve patients’ QoL and reduce the biopsychosocial impact of FMS.

### Study population

Patients with an active FMS diagnosis (International Classification of Diseases-10 codes: M79.0, M79.7) (38) within the GTTE health region were shortlisted from the eCAP system and recruited by telephone to participate in the study. Furthermore, the included individuals were all adults (over 18 years) with Catalan or Spanish language skills, a phone number, and no record of a psychotic episode, intellectual impairment, severe depression and personality disorder, auto/hyperaggressive behaviour, or consumption of psychoactive substances as noteworthy comorbidities. Informed written consent was required for participation in the study, and anonymous data management was ensured for data analysis and publication.

Individual-based random allocation to intervention and control (waiting list) groups was stratified by primary care centres due to the sociodemographic variation throughout the territory. Randomized lists were created by the Efron procedure (39) in advance and delivered to the researchers before patients’ visits for baseline data collection. Both researchers and patients were kept blind during the first encounter and until the next contact call, when patients were informed if they were recruited for the intervention or control group. Intervention groups included 8–15 participants. Patients in the control group were offered to receive the MCI after their follow-up period in addition to the UCC.

### Data collection and data sources

The MCI programme has been conducted since April 2017 through a multi-stage strategy, including 5 waves until January 2020, when the sample size for the RCT study was achieved (Table SI). The study follow-up ended in March 2021. This implementation scheme was based on the availability of patients with FMS who were willing to participate in the study and personnel in the different primary care centres. Due to the outbreak of SARS-CoV-2 (COVID-19), online data collection surveys and phone calls were implemented in addition to face-to-face interviews. The collected data were registered into a software application for FMS within the ICS health digital system.

*Sociodemographic and clinical variables.* Sociodemographic and clinical variables were extracted from the eCAP system at baseline.

*Health outcomes.* Based on the results obtained from the SF-36v2 questionnaire (40) (Optum, Inc., license number QM048943), quality-adjusted life years (QALYs) were calculated using the SF-6Dv2 (41) instrument weighted for the Spanish population (42) (QualityMetric, non-commercial license, order 240835D). Health-related QoL measurements were collected at baseline, immediately post-intervention, and 6 and 12 months after the programme ended. However, only pre- and 12-month post-intervention data were included in this study sample.

*Cost outcomes.* Cost outcomes and data sources are detailed in Table SII. Medical expenditure (direct costs) and productivity losses (indirect costs) were collected during the 12 months before and after the administration of the MCI programme. Costs were estimated in euros (€), corresponding to current prices in 2021, according to official Spanish service prices (43).

The estimation of medical expenditure included healthcare services in primary care and the regional Hospital de Tortosa Verge de la Cinta, whose costs were obtained by multiplying the number of services delivered per unit cost (Table SII). Only prescribed drugs (all types), partially publicly financed, were included in pharmaceutical expenditure. Costs were calculated by multiplying the prescribed number of administration days with its correspondence cost of treatment per day (CTD) linked to the drug national code and according to the official final consumer prices in September 2021 (44). All prices were used including taxes in accordance with the societal perspective outlined in the study.

Furthermore, full sick-leave days prescribed by the GP were endorsed as productivity losses. Indirect costs were estimated by multiplying the number of sick-leave days by the total daily mean wages before tax for 2021 in Catalonia, obtained from the Spanish National Statistics Institute (NSI) (45). As an indicator of the weighted price for the social costs, this included regular and extra payments, part-time and full-time working schedules, and all activity sectors (industry, construction, and all services except housework). According to the Spanish General Law of Social Security (Law 20/2014; Royal Legislative Decree 8/2015) (46), sick-leave days conceptually and operationally refer to “temporary disability” that entails absenteeism from work due to short-term common or work-related illness, based on a GP’s criteria. Even though the temporary disability payments are shared between the National Social Security System, the Social Security Mutual Society Partner and the employers (47), depending on the number of sick-leave days, we considered the same price weight for all days due to the societal perspective of the study.

Lastly, the MCI cost per participant was estimated based on the actual professionals’ services expenditure and the time (in h) dedicated to the programme. As the staff’s payment was based on working hours regardless of the number of participants, a mean of 10 patients per group was adopted to estimate the individual cost of the intervention (Table SII).

### Statistical analysis

Given the pragmatic nature of the study, the sample size attained and the moderate dropout level (25%), the analysis included complete cases in the follow-up. Hence, only those individuals who answered the pre-post intervention questionnaires, and whose data for estimating costs was available in the eCAP system, were included in the analysed sample. Therefore, non-included cases encompassed those individuals who either dropped out from the MCI or were lost during the study follow-up. This analytical strategy intended to capture real-world data, including individuals who attended the MCI regardless of the number of sessions attended. Accordingly, the study randomization was preserved, different therapeutic adherences were included, and the sample size was monitored carefully to ensure study rigour.

Using R Studio software (48), a description of the sample was carried out by including a bivariate analysis of the sociodemographic and clinical variables and independence tests (Student's *t*-test, Pearson’s χ^2^ test, and Fisher’s exact test) to assess the homogeneity of the sample at baseline. Furthermore, QALYs and costs per major component were statistically described for the pre- and post-intervention study periods, including means, percentile bootstrap confidence intervals, absolute mean differences and *p*-values. Student's *t*-test, Wilcoxon signed-rank and rank-sum tests were applied to compare the study groups’ paired and independent mean differences.

Thirdly, the incremental cost-utility ratio (ICUR) was calculated based on crude and adjusted, seemingly unrelated, regression (SUR) models (49) (systemfit R package; R Development Core Team), which estimate a set of equations for costs and QALYs jointly, assuming the correlation of the error terms. This statistical method has been implemented in previous CUA studies (26). Furthermore, bootstrap intervals were estimated for the difference in incremental costs and QALYs between the study groups in order to accommodate for the skewed distribution of these variables.

The ICUR results from the quotient between the differences in the total mean costs and the QALYs between the MIC programme and the UCC adjusted by the pre-intervention values. The result is interpreted according to the cost-effectiveness plane, where 4 decision scenarios are possible across the quadrants. In 2 of these scenarios, the ICUR presents less room for doubt, as the incremental cost-effect of the new intervention is either dominated (quadrant II: north-west) by the standard practice, for proving to be more costly and less effective, or dominant (quadrant IV: south-east) concerning the latest for reducing costs and improving health. In the remaining quadrants, the interpretation relies on the threshold that the founder is willing to pay for a QALY gained, since either the new intervention is more costly, but more effective (quadrant I: north-east) or cost-saving, but less effective, compared with its alternative (quadrant III: south-west). In this regard, it is recommended that everything that falls below the threshold should be accepted and rejected otherwise (50).

Finally, 1-way and 2-way deterministic sensitivity analyses (SA) were conducted to evaluate the robustness of the results (51, 52). The SA criteria entailed primary care cost variations (GP and nursing) based on the regional weighted health expenditure from 2021 (Table SIII), provided by the Catalonian Primary Care Services Information System (SISAP), and the exclusion from the sample of those participants with a session attendance < 66% (with a minimum participation of 8 out of 12 sessions). The latter was intended to assess the implementation scheme of the MCI programme.

## RESULTS

**Fig. 1:**
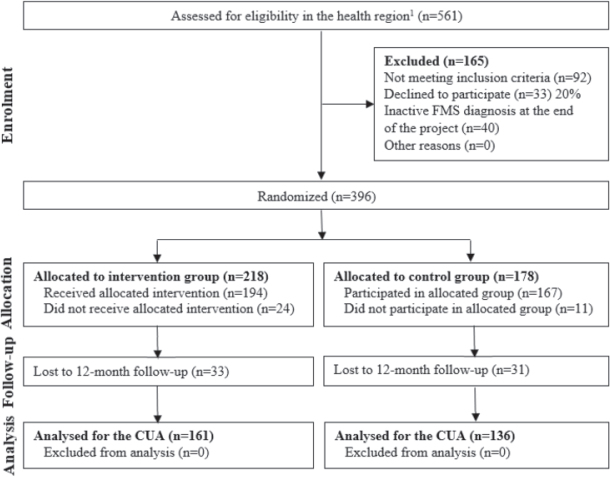
Sample flow diagram. CUA: cost-utility analysis; FMS: fibromyalgia syndrome. ^1^Patients with an active FMS diagnosis in their digital medical record system (eCAP) in Gerencia Territorial Terrres de L’Ebre, Catalonia, Spain.

A total of 297 individuals were included in the analysis, 161 and 136 in the intervention and control groups, respectively, which meets the estimated sample size for this study (260 individuals, 130 per study arm) (53). As expected for pragmatic RCTs, missing data entailed 25% of the initial randomized sample (*n* = 396), including those who dropped out of the study before or during the programme course (9%) and those who were lost during follow-up (18%) (Fig. 1). Overall, these non-included individuals showed a relatively similar sociodemographic and clinical profile compared with the included cases (Table SIV).

Table I displays the distribution of the sociodemographic and clinical characteristics of the total sample and according to study groups at baseline, which proved to be comparatively homogeneous. Notably, the sample is composed principally of women with a low education level on the whole. Statistical differences among the study groups showed that more participants in the MCI programme reported not having achieved basic schooling. Regarding work-life, approximately 47% of the sample was out of the labour force, 35% was employed, and 10% was unemployed at the beginning of the programme. Moreover, 63% of the participants were manual workers.

**Table I:** Distribution of the sociodemographic and clinical characteristics of the sample

	Total N = 297	Intervention *n* = 161	Control *n* = 136	*p*-value
*Sociodemographic characteristics*				
Sex, *n* (%)				
Female	288 (97)	157 (98)	131 (96)	0.7367
Male	9 (3)	4 (2)	5 (4)	
Age, years, mean (SD)	58.18 (10.5)	57.25 (10.15)	59.29 (10.84)	0.0957
Median (min–max)	58 (25–83)	58 (33–79)	59 (25–83)	
Birth country, *n* (%)				
Spain	290 (98)	159 (99)	131 (96)	0.253
Other	7 (2)	2 (1)	5 (4)	
Education, *n* (%)				
None	52 (18)	36 (22)	16 (12)	0.0025**
Primary	137 (46)	71 (44)	66 (49)	
Secondary	63 (21)	34 (21)	29 (21)	
Tertiary	20 (7)	4 (2)	16 (12)	
Missing	25 (8)	16 (10)	9 (7)	
Marital status, *n* (%)				
Married	208 (70)	119 (74)	89 (65)	0.1
Divorced	35 (12)	16 (10)	19 (14)	
Single	13 (4)	6 (4)	7 (5)	
Widow/er	17 (6)	5 (3)	12 (9)	
Missing	24 (8)	15 (9)	9 (7)	
Living alone, *n* (%)	25 (8)	6 (4)	19 (14)	0.0026**
Living with partner, *n* (%)	209 (70)	117 (73)	92 (67)	0.3733
Living with partner and children, *n* (%)	71 (24)	37 (23)	34 (25)	0.685
Living with partner, children and parents, *n* (%)	3 (1)	2 (1)	1 (1)	1
Living with others, *n* (%)	11 (4)	4 (3)	7 (5)	0.3558
Working condition, *n* (%)				
Employed	105 (35)	59 (37)	46 (34)	0.2448
Unemployed	29 (10)	13 (8)	16 (12)	
Retired	70 (24)	32 (20)	38 (28)	
Disabled	23 (8)	16 (10)	7 (5)	
Homemaker	46 (15)	26 (16)	20 (15)	
Missing	24 (8)	15 (9)	9 (7)	
Occupational class, *n* (%)				
I: Professionals	23 (8)	7 (4)	16 (12)	0.1509
II: Intermediate occupations	21 (7)	12 (7)	9 (7)	
III: Skilled non-manual workers	36 (12)	21 (13)	15 (11)	
IVa: Skilled manual workers	33 (11)	21 (13)	12 (9)	
IVb: Other manual workers	153 (52)	82 (51)	71 (52)	
Missing	31 (10)	18 (11)	13 (10)	
*Clinical characteristics*				
Years since FMS diagnosis, mean (SD)	7.05 (6)	6.83 (6.15)	7.30 (5.83)	0.5026
Median (min–max)	6 (0–39)	6 (0–39)	6 (0–25)	
Having a family history of FMS, *n* (%)	84 (28)	46 (29)	38 (28)	1
Physical trigger factor, *n* (%)	58 (19)	32 (20)	26 (19)	0.8844
Psychological trigger factor, *n* (%)	83 (28)	43 (27)	40 (29)	0.6068
Physical activity as trigger factor, *n* (%)	75 (25)	37 (23)	38 (28)	0.35
Stress as trigger factor, *n* (%)	140 (47)	75 (47)	65 (48)	0.9072
Total symptoms, mean (SD)	5.95 (2.80)	5.74 (2.84)	6.21 (2.74)	0.1526
Median (min–max)	6 (0–12)	6 (0–12)	7 (0–12)	
HADS scale, *n* (%)				
≤ 14	51 (17)	22 (14)	29 (21)	0.1486
> 14 ≤ 22	102 (34)	54 (33)	48 (35)	
> 22 ≤ 42	141 (48)	83 (52)	58 (43)	
Missing	3 (1)	2 (1)	1 (1)	
FIQR total score, mean (SD)	66.16 (18.67)	65.08 (19.47)	67.44 (17.67)	0.2796
Median (min–max)	68.25 (0–97.5)	67.83 (0–96.33)	69.09 (0–97.5)	
Comorbidities, *n* (%)				
Yes	183 (62)	90 (56)	93 (68)	0.0313*
No	114 (38)	71 (44)	43 (32)	
Attendance to MCI programme, mean (SD)	N/A	9.7 (2.32)	N/A	N/A
Median (min–max)	N/A	10 (1–12)	N/A	
Missing	N/A	5	N/A	

FMS: fibromyalgia syndrome; HADS: Hospital Anxiety and Depression Scale; FIQR: Revised Fibromyalgia Impact Questionnaire; MCI: multicomponent intervention; N/A: not applicable; SD: standard deviation; sig: significance; min: minimum; max: maximum.

* sig. ≤ 0.05.

** sig. ≤ 0.01.

***sig. ≤ 0.001.

Clinically, the variable “total symptoms” summarizes both physical and psychological common signs in FMS. Individuals were experiencing a mean of 6 symptoms at baseline, including attention and memory disturbances, restless sleep, paraesthesia, low back pain and fatigue, among the most frequently reported. Regarding anxiety and depression, the results from the Hospital Anxiety and Depression Scale (HADS) (54, 55) show that approximately 48% of the sample had the highest anxiety and depression levels. In addition, as the mean score from the Revised Fibromyalgia Impact Questionnaire (FIQR) (56, 57) was close to 70 (out of 100), it suggests that this condition severely afflicted the participants. Comorbidities were explored in the eCAP following the literature (58), which indicates a list of potential diagnoses that share similar symptomatology with FMS, especially in women (Table SV). The presence of comorbidities was found in over 60% of the total sample, and it showed statistical differences between the study groups as controls had more registered simultaneous medical conditions.

Lastly, the programme achieved a high level of participation, with a mean session attendance of over 80% and only 12.4% of the sample with fewer than 8 sessions (< 66%) listed.

Costs and health outcomes for the pre- and post-intervention periods according to study groups are described in Table II. Primary care and prescribed drug components contributed the most to direct costs at baseline, accounting for more than 86% in the intervention group and 67% in the control group. Nonetheless, these values remained relatively constant for the MCI participants in the post-intervention period, whereas they increased by 28% for the controls, particularly in primary care services. Moreover, specialized medical care and diagnostic imaging tests had the lowest contributions to direct costs (approximately 10% and 3%, respectively). They did not show significant differences between the study groups, either pre- or post-intervention. Even though the results were not statistically significant, direct costs showed a growing trend in the intervention group while they decreased in the controls. The post-intervention comparison of direct costs between the study groups indicated potential cost-saving in favour of the programme, non-significant nonetheless.

**Table II:** Descriptive statistics of costs (per major components) and health outcomes by study groups

		Intervention Mean [Bootstrap 95% CI]	Control Mean [Bootstrap 95% CI]	Difference Absolute mean diff. [Bootstrap 95% CI]	Independent *p*-value
**Cost outcomes**					
*Primary care*	pre-intv.	**967.18** [856.96 ; 1,083]	**1,075.13** [895.65 ; 1,338.5]	–107.95 [–389.34 ; 111.94]	0.6244
	post-intv.	↑ **1,030.78** [912.27 ; 1,155.78]	↑ **1,181.04** [1045.49 ; 1327.3]	–150.26 [–343.67 ; 37.35]	0.1124
	*paired p-value*	*0.4683*	*0.02813* *		
***Specialized*** *medical care (Hospital)*	pre-intv.	**220.73** [167.19 ; 279.75]	**264.78** [194.36 ; 340.9]	–44.05 [–137.16 ; 47.42]	0.7217
	post-intv.	↑ **222.07** [163.82 ; 288.2]	↓ **262.28** [187.46 ; 342.07]	–40.21 [–141.78 ; 60.89]	0.674
	*paired p-value*	*0.6658*	*0.3446*		
*Diagnostic imaging tests*	pre-intv.	**74.24** [54.77 ; 95.68]	**60.13** [45.77 ; 75.27]	14.11 [–10.47 ; 39.31]	0.4791
	post-intv.	↓ **66.56** [47.53 ; 87.69]	↑ **66.08** [47.47 ; 87.2]	0.49 [–27.79 ; 29.24]	0.7444
	*paired p-value*	*0.1235*	*0.9511*		
*Prescribed drugs*	pre-intv.	**882.38** [734.25 ; 1,050.04]	**1,066.96** [866.91 ; 1,291.35]	–184.59 [–458.17 ; 76.37]	0.1297
	post-intv.	↑ **959.34** [760.9 ; 1,192.68]	↓ **917.68** [778.11 ; 1,069.75]	41.66 [–213.72 ; 326.36]	0.2425
	*paired p-value*	*0.8128*	*0.05914*		
**Direct costs**	pre-intv.	**2,144.53** [1,897.13 ; 2,404.08]	**2,467.01** [2,131.04 ; 2,851.41]	–322.48 [–787.96 ; 103.11]	0.2794
	post-intv.	↑ **2,278.75** [1,971.05 ; 2,611.04]	↓ **2,427.07** [2,163.17 ; 2,699.01]	–148.33 [–556.52 ; 276.39]	0.1211
	*paired p-value*	*0.4189*	*0.5754*		
**Indirect costs**	pre-intv.	**3,290.95** [2,166.04 ; 4,557.69]	**3,176.46** [1,964.77 ; 4,495.37]	114.49 [–1,605.04 ; 1,865.02]	0.9412
	post-intv.	↓ **2,206.50** [1,321.36 ; 3,202.14]	↓ **2,112.47** [1,175.21 ; 3,214.24]	94.02 [–1,288.54 ; 1,469.76]	0.5942
	*paired p-value*	*0.1041*	*0.03381* *		
**TOTAL COSTS**	pre-intv.	**5,435.48** [4,241.9 ; 6,714.72]	**5,643.47** [4,291.46 ; 7,116.44]	–207.99 [–2,120.25; 1,670.63]	0.5593
	post-intv.	↓ **4,545.24** [3,568.23 ; 5,630.57]	↓ **4,539.54** [3,499.36 ; 5,734.02]	5.7 [–1,503.05 ; 1,552.76]	0.6011
	*paired p-value*	*0.8263*	*0.2599*		
**Health outcome**					
QALYs	pre-intv.	**0.22** [0.18 ; 0.25]	**0.21** [0.17 ; 0.25]	0.01 [–0.04 ; 0.06]	0.7706
	post-intv.	↑ **0.31** [0.27 ; 0.35]	↓ **0.19** [0.14 ; 0.23]	0.13 [0.07 ; 0.18]	0,0264*
	*paired p-value*	*0.00000263* ***	*0.3467*		

The bootstrap confidence intervals were computed using 10,000 bootstrap replications. All costs are presented in euros (€) according to 2021 prices.

diff: differences; intv.: intervention; QALYs: quality-adjusted life years; *paired p-value*: intra-group mean comparison; *independent p-value*: inter-group mean comparison; sig.: significance; 95% CI: 95% confidence interval.

* sig. ≤ 0.05.

**sig. ≤ 0.01.

*** sig. ≤ 0.001.

Not surprisingly, indirect costs represented more than 50% of the total costs. In the pre-post intervention comparison, a decreasing shift was found in the costs of productivity losses in both groups separately. However, no statistical differences arose between the study groups.

Overall, total costs decreased for both study groups post-intervention, yet not significantly. In addition, the differences between treatments were not substantial.

Regarding the gain of QALYs, a significant improvement was detected in the intervention group and compared with the standard practice post-intervention.

**Fig. 2:**
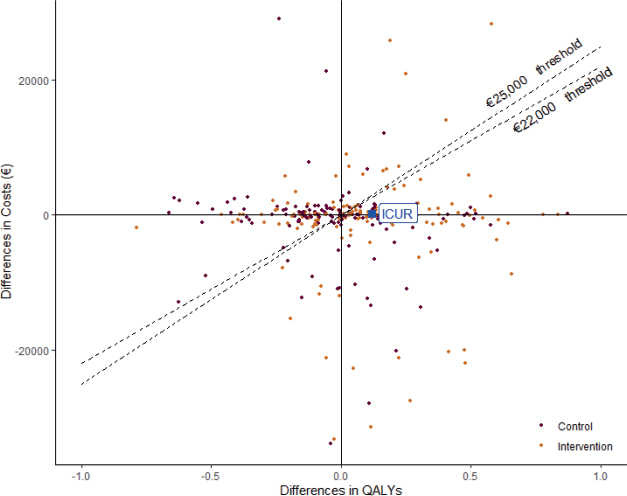
Cost and effect differences by study group and cost-utility ratio (ICUR). The incremental cost-utility ratio (ICUR) in Fig. 2 corresponds to the adjusted model € 851,67/quality-adjusted life years (QALY) from Table III.

Table III presents the mean differences in the total costs and the QALYs, pre- and post-intervention, for each study group and the incremental outcome between them with the respective estimated ICURs. The crude model showed an incremental cost between the study groups of €213.69 and an incremental effect of 0.12 QALYs per year, resulting in an ICUR of €1,780.75 per QALY gained. Nonetheless, this estimation decreases when being adjusted by sociodemographic and clinical variables, resulting in an incremental cost of € 102.20 but with the same incremental effect and an ICUR of €851.67 per QALY gained. In both cases, ICUR is situated in the first quadrant of the cost-effectiveness plane, which means that the MCI programme raises the cost of treating patients with FMS, but with a significant health improvement. Despite the cost-increasing result, the confidence intervals showed no significant differences, and the ICUR falls way below the cost-effectiveness threshold, as shown in Fig. 2. According to the literature, the cost-effectiveness threshold has been estimated between €22,000 and €25,000 for the Spanish National Health System (NHS) (59), even though it could also increase to €30,000.

**Table III:** Incremental costs and effects pre-post intervention by study group

	Cost difference pre-post intervention Mean [Bootstrap 95% CI]	Effect difference pre-post intervention Mean [Bootstrap 95% CI]
Intervention	–890.24 [–2,098.77 ; 242.55]	0.09 [0.06 ; 0.14]
Control	–1103.93 [–2,203.64 ; –58.29]	–0.02 [–0.06 ; 0.02]

	Δ Incremental total costs Mean [Bootstrap 95% CI]	Δ Incremental effect Mean [Bootstrap 95% CI]

Intervention vs Control (crude model)	213.69 [–1,394.94 ; 1,822.32]	0.12 [0.06 ; 0.18]
ICUR (€/QALY)	1,780.75 (quadrant I)	
Intervention vs Control (adjusted model)	102.20 [–1,460.92 ; 1,630.59]	0.12 [0.06 ; 0,18]
Adjusted ICUR^a^ (€/QALY)	851.67 (quadrant I)	

a Incremental cost-utility ratio (ICUR) adjusted by the following covariates: age, education level, living alone, years since diagnosis, reported symptoms, having a family history of fibromyalgia, presence of comorbidities, Hospital Anxiety and Depression Scale (HADS) and Revised Fibromyalgia Impact Questionnaire (FIQR) total scores. QALY: quality-adjusted life year; 95% CI: 95% confidence interval. Note: the bootstrap confidence intervals were computed using 10,000 bootstrap replications.

The 1-way deterministic SA included variations in primary care costs, specifically on GP and nurse services, due to its large contributions to total costs. The results in Table IV support the robustness of the study findings with a crude ICUR of €1,665.27 per QALY gained and an adjusted ICUR of €644.39. These new results reduced the previous estimations and were also below the cost-effectiveness threshold.

**Table IV:** One-way and 2-way deterministic sensitivity analyses

	Cost difference pre-post intervention Mean [Bootstrap 95% CI]		Effect difference pre-post intervention Mean [Bootstrap 95% CI]	
	One-way SA	Two-way SA	One-way SA	Two-way SA
Intervention	–880.03 [–2,099.84; 267.46]	–1,165.75 [–2,095.64; 254.57]	0.10 [0.06 ; 0.14]	0.11 [0.06 ; 0.14]
Control	–1,078.19 [–2,191.61; –28.35]	–1,078.19 [–2,398.75; 32.49]	–0.02 [–0.06 ; 0.02]	–0.02 [–0.07 ; 0.03]

	Δ Incremental total costs Mean [Bootstrap 95% CI]		Δ Incremental effect Mean [Bootstrap 95% CI]	
	One-way SA	Two-way SA	One-way SA	Two-way SA

Intervention vs Control (crude model)	198.159 [–1,435.98; 1,786.77]	–87.55 [–1,445.32; 1,954.16 ]	0.12 [0.06 ; 0.17]	0.13 [0.06 ; 0.18]
ICUR (€/QALY)	1,665.27 (quadrant I)	–668.21 (MCI Dominant)		
Intervention vs Control (adjusted model)	77.41 [–1,490.07; 1,614.95]	–272.29 [–1,546.14; 1,651.31]	0.12 [0.06 ; 0.18]	0.13 [0.05 ; 0.18]
Adjusted ICUR^1^ (€/QALY)	644.39 (quadrant I)	–2,141.31 (MCI Dominant)		

Incremental cost-utility ratio (ICUR) adjusted by the following covariates: age, education level, living alone, years since diagnostic, reported symptoms, having a family history of fibromyalgia, presence of comorbidities, Hospital Anxiety and Depression Scale (HADS) and Revised Fibromyalgia Impact Questionnaire (FIQR) total scores.

QALY: quality-adjusted life year; SA: sensitivity analyses; 95% CI: 95% confidence interval. Note: The bootstrap confidence intervals were computed using 10,000 bootstrap replications.

As expected, the outcomes of the 2-way deterministic SA (Table IV), revealed a dominant scenario for the MCI programme when not accounting for the 12.4% of participants with < 66% of session attendance. Excluding those 20 individuals from the intervention group resulted in a new crude ICUR of – €668.21 per QALY gained, which entails cost savings and health enhancement. Furthermore, the adjusted model of the incremental costs and effect differences triggered €2,141.31 of savings per QALY gained, which confirmed and increased the programme’s dominance.

## DISCUSSION

This study presents a trial-based CUA conducted on a multidisciplinary intervention for patients with FMS in Spain, which proved cost-effective compared with usual practice. The results show that the proposed programme improves patients’ QALYs significantly compared with the control group, and its incremental costs do not exceed the cost-effectiveness threshold, with the potential for cost-savings when the MCI was administrated to al least 66%.

The incremental cost-effectiveness ratio (ICER) is the most popular tool for reporting CUAs. Notably, alternatives are gaining relevance in the economic evaluation scene. Paulden (60, 61) claims it may be time to replace the ICER with measurements such as net benefit. The author advocates for its simplicity in calculating and interpreting the cost-effectiveness of each healthcare strategy individually and the probabilistic assessment of the uncertainty of the results in contrast to the pairwise ICER. Nonetheless, these methodological advantages are particularly gainful when comparing more than 2 alternatives, which is not the case in this study. In addition, the use of the ICER is still in force in Spain and is supported by international guidelines (62, 63).

The singularity of the CUA is the use of the QALYs. This metric allows incorporation of patients’ self-reported health perception into consideration when evaluating the benefits of a new medical intervention, and it allows comparisons between strategies with different health outcomes. In agreement with the literature, the estimated QALYs showed a decreased lower threshold limit compared with the results we would have obtained if using the utility model for the UK. As reported by Abellán Perpiñán (42), the Spanish utility model produces considerably smaller metrics compared with the one from the UK, which explains the modest margin found in this study between 0.18 and 0.35. However, the MCI yielded statistically significant improvements in QALYs compared with the control group, which provides evidence in its favour.

Contrary to expectations, no statistically significant differences in direct and indirect costs were observed between the study groups. These findings are less surprising if we consider that waves 4 and 5 of the programme, representing 44.2% of the sample, had their post-intervention data collected throughout 2020 and the beginning of 2021 during the most challenging times of the COVID-19 (SAR-CoV-2) pandemic. According to the results of an *ad hoc* survey administered to these subgroups in June and July 2020 (data not published), it was observed that 48% of the respondents (44% in the intervention group and 56% in the control group) reported an adverse effect on the FMS symptoms after the first wave of the pandemic. Therefore, it is highly likely that both costs and health outcomes could have been negatively impacted by this critical event in both study groups. Studies have described severe consequences for patients with FMS associated with the pandemic measurements, including sleep disturbances, poor QoL, pain increase, and psychological distress, particularly in those countries with strict and long periods of lockdown, such as Italy, France or Spain (64–66). A scoping review conducted in Spain has evidenced the impact on the physical and mental health of patients with chronic pain and stressed the need for data on medical consultations and the development of preventive protocols (67). We would encourage researchers to examine this phenomenon for the Spanish FMS community in terms of the use of health services and medication consumption, among other relevant factors. In any case, the proposed MCI programme has proved cost-effective beyond the potential repercussions of the COVID-19 pandemic. In addition, future research could explore other potential influential factors in resource utilization among patients with FMS, including (but not exclusively) chronic use of medication and associated comorbidities.

The results of the current study are consistent with previous studies from the last 10 years showing a dominant pattern of non-pharmacological intervention strategies for FMS compared with the usual clinical practice in Spain (23–28). The findings would suggest that patients with FMS and society will benefit from incorporating new holistic approaches, such as, but not limited to, health education, CBT, mindfulness, relaxation techniques, and physical rehabilitation. D’Amico et al. (28) recently conducted a pilot RCT in the Spanish healthcare system to compare attachment-based compassion therapy with relaxation. Their results have shown promise for future psychotherapy approaches in treating FMS, highlighting the potential gains for the public sector. We consider that the current study broadens these findings to society as a whole.

Lastly, the presented SA supports the robustness of the study results. It particularly demonstrates the benefits of implementing the MCI programme in a real-cost scenario and the relevance and benefits of covering the programme session scheme as planned. As discovered through qualitative research within this project, the programme’s success relies not only on implementing intervention techniques, but also on the process and group effect it triggers (68). The finding that increasing patient participation leads to better outcomes could be taken as an incentive for supporting complex interventions and preventing them from being shortened in length and professionals involved in contexts of limited resources. The discovery that enhanced patient involvement contributes to improved outcomes serves as motivation to advocate for the preservation of comprehensive interventions and to discourage their shortening, especially in terms of both length and human resources. Furthermore, a follow-up post-MCI could benefit the implementation of the programme learnings to boost lifestyle changes.

### Strengths and limitations

This study is novel in conducting a pragmatic trial-based CUA in the Spanish public healthcare sector on a MCI programme for patients with FMS and achieving the described sample size. Multiple challenges were overcome during this process, including the multicentre strategy throughout the healthcare region, personnel training, patient follow-up monitoring, and the outbreak of COVID-19.

Regarding the study design, economic evaluations conducted alongside RCTs are described in the literature as piggyback evaluations (69), which carry several potential drawbacks. One of them is the sample size estimation, as it generally responds to an outcome variable that does not meet the needs of a health economic study. Nonetheless, in this case, the sample size of the linked RCT was estimated based on the expected differences between the study groups from the SF-36v2 questionnaire. Accordingly, 260 subjects (130 per study arm) were estimated to detect a difference of at least 5 points, assuming an α error of 0.05, a β error of 0.05 (bilateral contrast), and a 20% dropout rate. As this study reached the calculated sample, a complete case analysis strategy was implemented. In addition, as the number of non-included individuals was according to expectations and showed a fairly similar sociodemographic and clinical profile compared with the rest of the sample, there is no indication to assume that using a different analytical approach by imputing missing data would lead to meaningfully different results. Even though complete case analysis faces a potential loss of valuable data, it also preserves real-world information through a simple and robust method.

Concerning the cost outcomes used for this study, none of the direct medical cost components could be specifically identified for the diagnosis and attention of FMS, which could have led to an overestimation of the real costs. Nevertheless, given the societal perspective of the study, this obstacle may have been offset by the lack of data about over-the-counter drugs and out-of-pocket health services consumption, especially for rehabilitation and psychological help. Given the non-inclusion of information on non-health costs (such as administrative costs, training, waiting time, and travelling, among others) or care-given costs, the study findings should not be over-interpreted in terms of societal benefits. However, the primary care cost variations included in the SA were weighted according to the regional health expenditure from 2021, including additional operating expenses. Future research must address these estimates in more detail.

Performing a SA by excluding individuals with < 66% session attendance pursued implementation assessment purposes. Given the limited resources of the public health system, health programmes may tend to be reduced and simplified to meet other priorities. Even though not all patients would reach this level of participation in the MCI in practice, this methodological decision on the SA only excluded a small portion of the sample (12.4%) and entailed a standard therapeutic adherence expectation.

Likewise, the human capital approach may tend to overestimate productivity losses since work replacement is not considered as in another method, such as the friction cost approach. However, this methodological limitation could have been outweighed by the 47% of the sample who were no in employment (pensioners and homemakers) whose loss of contribution to society could not be captured. In addition, productivity losses were only accountable for the registered sick-leave days in the eCAP system, and, unfortunately, no data were available regarding publicly funded disability pension (DP). However, patients with FMS are rarely granted DP in Spain unless diagnosed with another more severe disabling disease.

Finally, the study findings are not generalizable beyond the assessed regional population, even though the proposed MCI may serve as an example to adapt the programme to other national or international contexts.

### Conclusion

The MCI was found to be cost-effective compared with the usual clinical practice for patients with FMS. The obtained incremental costs per QALY gained for those attending the MCI did not exceed the cost-effectiveness threshold compared with the control group. Furthermore, these results were confirmed by the sensitivity analysis, showing promise for implementation. Hence, the study results support strengthening the standard practice for FMS with the proposed MCI programme in regional primary care settings. These findings provide decision-makers with evidence to reinforce the treatment strategies for FMS in the public healthcare system and its efficiency.

## References

[1] Giorgi V, Sirotti S, Romano ME, Marotto D, Ablin JN, Salaffi F, et al. Fibromyalgia: one year in review 2022. Clin Exp Rheumatol 2022; 40: 1065–1072. DOI: 10.55563/clinexprheumatol/if9gk2 35748720

[2] Wolfe F, Walitt B, Perrot S, Rasker JJ, Häuser W. Fibromyalgia diagnosis and biased assessment: sex, prevalence and bias. PLoS One 2018; 13: 1–14. DOI: 10.1371/journal.pone.0203755 PMC6136749 30212526

[3] Pasqual Marques A, Espírito Santo A de S, Berssaneti AA, Matsutani LA, King Yuan SL. Prevalence of fibromyalgia: literature review update. Rev Bras Reumatol 2017; 57: 356–363. DOI: 10.1016/j.rbre.2017.01.005 28743363

[4] Font Gayà T, Bordoy Ferrer C, Juan Mas A, Seoane-Mato D, Álvarez Reyes F, Delgado Sánchez M, et al. Prevalence of fibromyalgia and associated factors in Spain. Clin Exp Rheumatol 2020; 38: 47–52. 31928589

[5] Ursini F, Ciaffi J, Mancarella L, Lisi L, Brusi V, Cavallari C, et al. Fibromyalgia: a new facet of the post-COVID-19 syndrome spectrum? Results based survey from a web-based survey. RMD Open 2021; 7: e001735. DOI: 10.1136/rmdopen-2021-001735 34426540 PMC8384499

[6] Wolfe F, Smythe HA, Yunus MB, Bennett RM, Bombardier C, Goldenberg DL, et al. The American College of Rheumatology 1990 Criteria for the Classification of Fibromyalgia. Report of the Multicenter Criteria Committee. Arthritis Rheum 1990; 33: 160–172. DOI: 10.1002/art.1780330203 2306288

[7] Wolfe F, Clauw DJ, Fitzcharles MA, Goldenberg DL, Katz RS, Mease P, et al. The American College of Rheumatology preliminary diagnostic criteria for fibromyalgia and measurement of symptom severity. Arthritis Care Res 2010; 62: 600–610. DOI: 10.1002/acr.20140 20461783

[8] Wolfe F, Clauw DJ, Fitzcharles MA, Goldenberg DL, Häuser W, Katz RL, et al. 2016 Revisions to the 2010/2011 fibromyalgia diagnostic criteria. Semin Arthritis Rheum 2016; 46: 319–329. DOI: 10.1016/j.semarthrit.2016.08.012 27916278

[9] de Tommaso M, Vecchio E, Nolano M. The puzzle of fibromyalgia between central sensitisation syndrome and small fiber neuropathy: a narrative review on neurophysiological and morphological evidence. Neurol Sci 2022; 43: 1667–1684. DOI: 10.1007/s10072-021-05806-x 35028777

[10] Spaeth M. Epidemiology, costs, and the economic burden of fibromyalgia. Arthritis Res Ther 2009; 11: 117. DOI: 10.1186/ar2715 19591654 PMC2714132

[11] Sicras-Mainar A, Rejas J, Navarro R, Milagrosa B, Morcillo A, Larios R, et al. Treating patients with fibromyalgia in primary care settings under routine medical practice: a claim database cost and burden of illness study. Arthritis Res Ther 2009; 11: R54. DOI: 10.1186/ar2673 19366441 PMC2688205

[12] Rivera J, Rejas J, Esteve-Vives J, Vallejo MA, Grupo ICAF. Resource utilisation and health care costs in patients diagnosed with fibromyalgia in Spain. Clin Exp Rheumatol 2009; 27: S39–S45. 20074438

[13] Thompson JM, Luedtke CA, Oh TH, Shah ND, Long KH, King S, et al. Direct medical costs in patients with fibromyalgia: cost of illness and impact of a brief multidisciplinary treatment program. Am J Phys Med Rehabil 2011; 90: 40–46. DOI: 10.1097/PHM.0b013e3181fc7ff3 20975520

[14] Vervoort VM, Vriezekolk JE, Olde Hartman TC, Cats HA, van Helmond T, van der Laan WH, et al. Cost of illness and illness perceptions in patients with fibromyalgia. Clin Exp Rheumatol 2016; 34: S74–S82. 26886404

[15] Cabo-Meseguer A, Cerda-Olmedo G, Trillo-Mata JL. Fibromyalgia: Prevalence, epidemiologic profiles and economic costs. Med Clin (Barc) 2017; 149: 441–448. DOI: 10.1016/j.medcli.2017.06.008 28734619

[16] Skaer TL. Fibromyalgia: disease synopsis, medication cost effectiveness and economic burden. Pharmacoeconomics 2014; 32: 457–466. DOI: 10.1007/s40273-014-0137-y 24504852

[17] Macfarlane GJ, Kronisch C, Dean LE, Atzeni F, Häuser W, FluB E, et al. EULAR revised recommendations for the management of fibromyalgia. Ann Rheum Dis 2017; 76: 318–328. DOI: 10.1136/annrheumdis-2016-209724 27377815

[18] Yin JH, Peng GS, Ro LS. Multidisciplinary approach to Fibromyalgia: What are we learning from updated evidence-based medicine? Acta Neurol Taiwan 2022 Jan 18 [online ahead of print]. 34918303

[19] Hernando-Garijo I, Jiménez-Del-Barrio S, Mingo-Gómez T, Medrano-De-La-Fuente R, Ceballos-Laita L. Effectiveness of non-pharmacological conservative therapies in adults with fibromyalgia: a systematic review of high-quality clinical trials. J Back Musculoskelet Rehabil 2022; 35: 3–20. DOI: 10.3233/BMR-200282 34180405

[20] Mascarenhas RO, Souza MB, Oliveira MX, Lacerda AC, Mendonça VA, Henschke N, et al. Association of therapies with reduced pain and improved quality of life in patients with fibromyalgia: a systematic review and meta-analysis. JAMA Intern Med 2021; 181: 104–112. https://doi:10.1001/jamainternmed.2020.5651 33104162 10.1001/jamainternmed.2020.5651 PMC7589080

[21] Cabral CMN, Miyamoto GC, Franco KFM, Bosmans JE. Economic evaluations of educational, physical, and psychological treatments for fibromyalgia: a systematic review with meta-analysis. Pain 2021; 162: 2331–2345. DOI: 10.1097/j.pain.0000000000002233 33605655

[22] Mccabe C. What is cost-utility analysis? 2009. [Accessed 15 Jun 2022]. Available from http://www.bandolier.org.uk/painres/download/whatis/What_is_cost-util.pdf

[23] Luciano JV, Sabes-Figuera R, Cardenosa E, Peñarrubia-María MT, Fernández-Vergel R, García-Campayo J, et al. Cost-utility of a psychoeducational intervention in fibromyalgia patients compared with usual care: an economic evaluation alongside a 12-month randomised controlled trial. Clin J Pain 2013; 29: 702–711. DOI: 10.1097/AJP.0b013e318270f99a 23328339

[24] Luciano JV, D'Amico F, Cerdà-Lafont M, Peñarrubia-María MT, Knapp M, Cuesta-Vargas AI, et al. Cost-utility of cognitive behavioral therapy versus U.S. Food and Drug Administration recommended drugs and usual care in the treatment of patients with fibromyalgia: an economic evaluation alongside a 6-month randomised controlled trial. Arthritis Res Ther 2014; 16: 1–17. DOI: 10.1186/s13075-014-0451-y PMC4203881 25270426

[25] Feliu-Soler A, Borràs X, Peñarrubia-María MT, Peñarrubia-María MT, Knapp M, Cuesta-Vargas AI, et al. Cost-utility and biological underpinnings of Mindfulness-Based Stress Reduction (MBSR) versus a psychoeducational programme (FibroQoL) for fibromyalgia: a 12-month randomised controlled trial (EUDAIMON study). BMC Complement Altern Med 2016; 16: 1–16. DOI: 10.1186/s12906-016-1068-2 26921267 PMC4769528

[26] Pérez-Aranda A, D’Amico F, Feliu-Soler A, McCracken LM, Peñarrubia-María MT, Andrés-Rodríguez L, et al. Cost–utility of mindfulness-based stress reduction for fibromyalgia versus a multicomponent intervention and usual care: a 12-month randomized controlled trial (EUDAIMON Study). J Clin Med 2019; 8: 1068. DOI: 10.3390/jcm8071068 31330832 PMC6678679

[27] Luciano J V, D’Amico F, Feliu-Soler A, McCracken LM, Aguado J, Peñarrubia-María MT, et al. Cost-utility of group acceptance and commitment therapy for fibromyalgia versus recommended drugs: an economic analysis alongside a 6-month randomized controlled trial conducted in Spain (EFFIGACT Study). J Pain 2017; 18: 868–880. DOI: 10.1016/j.jpain.2017.03.001 28342891

[28] D’amico F, Feliu-Soler A, Montero-Marín J, Peñarrubía-María MT, Navarro-Gil M, Van Gordon W, et al. Cost-utility of attachment-based compassion therapy (ABCT) for fibromyalgia compared to relaxation: a pilot randomised controlled trial. J Clin Med 2020; 9: 726. DOI: 10.3390/jcm9030726 32156065 PMC7141201

[29] Caballol Angelats R, Gonçalves AQ, Aguilar Martín C, Sol MCS, Serra GG, Casajuana M, et al. Effectiveness, cost-utility, and benefits of a multicomponent therapy to improve the quality of life of patients with fibromyalgia in primary care: a mixed methods study protocol. Med (United States) 2019; 98: e17289. DOI: 10.1097/MD.0000000000017289 PMC6799432 31593081

[30] Jönsson B. Editorial: ten arguments for a societal perspective in the economic evaluation of medical innovations. Eur J Heal Econ 2009; 10: 357–359. DOI: 10.1007/s10198-009-0173-2 19618224

[31] Kobelt G. Health Economics: an Introduction to Economic Evaluation. Vol 314. Third Edit. (Office of Health Economics (OHE), ed.). OHE Monograph 2013. DOI: 10.1136/bmj.314.7098.1916a

[32] Zwarenstein M, Treweek S, Gagnier JJ, Altman DG, Tunis S, Haynes B. Improving the reporting of pragmatic trials : an ex-tension of the CONSORT statement. BMJ 2008; 337: a2390. DOI: 10.1136/bmj.a2390 19001484 PMC3266844

[33] Husereau D, Drummond M, Augustovski F, de Bekker-Grob E, Briggs AH, Carswell C, et al. Consolidated Health Economic Evaluation Reporting Standards 2022 (CHEERS 2022) Statement: updated reporting guidance for health economic evaluations. Value Heal 2022; 25: 3–9. DOI: 10.1016/j.jval.2021.11.1351 35031096

[34] Craig P, Dieppe P, Macintyre S, Michie S, Nazareth I, Petticrew M. Developing and evaluating complex interventions: the new Medical Research Council guidance. Int J Nurs Stud 2013; 50: 587–592. DOI: 10.1016/j.ijnurstu.2012.09.010 23159157

[35] Rodríguez-Feijoó S, Rodríguez-Caro A. [Pharmaceutical copayment in Spain after the 2012 reform from the user’s perspective. Evidence of inequity?] Gac Sanit 2021; 35: 138–144 (in Spanish). DOI: 10.1016/j.gaceta.2019.09.009 31879054

[36] Celaya M, Ibáñez D, Laseca N, López A, Romero C, Valls E. Guia per a l’avaluació de la fibromiàlgia i de la síndrome de fatiga crònica. Barcelona: Direcció General d’Ordenació Professional i Regulació Sanitària; 2017. [accessed 2022 Jun 17]. Available from: http://hdl.handle.net/11351/5652

[37] Catalunya G de. Diari Oficial de la Generalitat de Catalunya. [accessed 2022 Mar 15]. Available from: https://dogc.gencat.cat/es

[38] World Health Organization (WHO). ICD-10: International Statistical Classification of Diseases and Related Health Problems: tenth revision. 2nd edn. Geneva: World Health Organization; 2004.

[39] Efron B. Forcing a Sequential Experiment to be balanced. Biometrika 1971; 58: 403–417. DOI: 10.2307/2334377

[40] Alonso J. Versión española de SF-36v2TM Health Survey © 1996, 2000 adaptada por J. Alonso y cols 2003. Heal Surv Published online 2003: 1-8. [accessed 2020 Jan 10]. Available from: http://www.ser.es/wp-content/uploads/2015/03/SF36_CUESTIONARIOpdf.pdf

[41] Rebollo P, Morís J, Ortega T, Valdés C, Ortega F. Estimating utility values for health status using the Spanish version of the SF-36. Validity of the SF-6D index vs EQ-5D. Med Clin (Barc) 2007; 128: 536–537. DOI: 10.1157/13101163 17433207

[42] Abellán-Perpiñán JM. Utilidades SF-6D Para España. Guía de Uso. Guía de uso 2012/8. Cátedra de Economía de la Salud. Universidad Pablo de Olavide. Sevilla: Consejería de Salud de la Junta de Andalucía; 2013.

[43] Gisbert, R and Brosa M. Spanish health costs and cost-effectiveness ratios Database: eSalud Published 2007 [accessed 2022 Jun 30]. Available from: http://www.oblikue.com/bddcostes/

[44] Ministerio de Sanidad de España. Boletín Oficial Del Estado Orden SND/1121/2020.; 2020 [accessed 2022 Jun 30]. Available from: https://www.boe.es/boe/dias/2020/11/28/pdfs/BOE-A-2020-15176.pdf

[45] Instituto Nacional de Estadística.Coste laboral por trabajador, comunidad autónoma, sectores de actividad [Labor wages per worker, autonomous community, sectors of activity][accessed Nov 15, 2021]. Available from https://www.ine.es/jaxiT3/Tabla.htm?t=60611

[46] Gobierno de España. Real Decreto 8/2015 por el que e aprueba el texto refundido de la Ley General de la Seguridad Social [Royal Decree 8/2015 approving the consolidated text of the General Social Security Law]. Boletín del Estado. Published online2015: 1–157 [accessed 2022 Mar 25]. Available from: http://www.boe.es/boe/dias/1994/06/29/pdfs/A20658-20708.pdf.

[47] Seguridad Social España. Prestación de Incapacidad Temporal [Temporary disability benefits] [accessed 2022 Jun 30]. Available from: https://www.seg-social.es/wps/portal/wss/internet/InformacionUtil/44539/44667#:~:text=En general%2C el pago lo,INSS o de la mutua.

[48] RStudio Team. RStudio: Integrated development environment for R. Published online 2022. Available from: http://www.rstudio.com/

[49] Mantopoulos T, Mitchell PM, Welton NJ, McManus R, Andronis L. Choice of statistical model for cost-effectiveness analysis and covariate adjustment: empirical application of prominent models and assessment of their results. Eur J Heal Econ 2016; 17: 927–938. DOI: 10.1007/s10198-015-0731-8 26445961

[50] Cohen DJ, Reynolds MR. Interpreting the results of cost-effectiveness studies. J Am Coll Cardiol 2008; 52: 2119–2126. DOI: 10.1016/j.jacc.2008.09.018 19095128 PMC2716087

[51] Andronis L, Barton P, Bryan S. Sensitivity analysis in economic evaluation: an audit of NICE current practice and a review of its use and value in decision-making. Health Technol Assess 2009; 13: iii, ix–xi, 1–61. DOI: 10.3310/hta13290 19500484

[52] Thabane L, Mbuagbaw L, Zhang S, Samaan Z, Marcucci M, Ye C, et al. A tutorial on sensitivity analyses in clinical trials: the what, why, when and how. BMC Med Res Methodol 2013; 13: 92. DOI: 10.1186/1471-2288-13-92 23855337 PMC3720188

[53] Arfuch VM, Aguilar Martín C, Berenguera A, Caballol Angelats R, Carrasco-Querol N, Serra GG, et al. Cost-utility analysis of a multicomponent intervention for fibromyalgia syndrome in primary care versus usual clinical practice: study protocol for an economic evaluation of a randomised control trial. BMJ Open 2021; 11: e043562. DOI: 10.1136/bmjopen-2020-043562 PMC7925862 33550259

[54] Vallejo MA, Rivera J, Esteve-Vives J, Rodríguez-Muñoz MF. [Use of the Hospital Anxiety and Depression Scale (HADS) to evaluate anxiety and depression in fibromyalgia patients]. Rev Psiquiatr Salud Ment 2012; 5: 107–114 (in Spanish). DOI: 10.1016/j.rpsm.2012.01.003 22854581

[55] Cabrera V, Martín-Aragón M, Terol MC, Nuñez R, Pastor MA. Hospital Anxiety and depression Scale (HADS) in fibromyalgia: sensitivity and specific analysis. Ter Psicológica 2015; 33: 181–193. DOI: 10.4067/S0718-48082015000300003

[56] Bennett RM, Friend R, Jones KD, Ward R, Han BK, Ross RL. The Revised Fibromyalgia Impact Questionnaire (FIQR): validation and psychometric properties. Arthritis Res Ther 2009; 11: R120. DOI: 10.1186/ar2783 19664287 PMC2745803

[57] Salgueiro M, García-Leiva JM, Ballesteros J, Hidalgo J, Molina R, Calandre EP. Validation of a Spanish version of the Revised Fibromyalgia Impact Questionnaire (FIQR). Health Qual Life Outcomes 2013; 11: 1–8. DOI: 10.1186/1477-7525-11-132 23915386 PMC3770447

[58] Valls Llobet C. [Differential diagnosis of pain and fibromyalgia]. Anu Psicol Univ Barcelona 2008; 39: 87–92 (in Spanish). DOI: 10.1344/%x

[59] Vallejo-Torres L, García-Lorenzo B, Serrano-Aguilar P. Estimating a cost-effectiveness threshold for the Spanish NHS. Health Econ 2018; 27: 746–761. DOI: 10.1002/hec.3633 29282798

[60] Paulden M. Why it’s time to abandon the ICER. Pharmacoeconomics 2020; 38: 781–784. DOI: 10.1007/s40273-020-00915-5 32390066

[61] Paulden M. Calculating and interpreting ICERs and net benefit. Pharmacoeconomics 2020; 38: 785–807. DOI: 10.1007/s40273-020-00914-6 32390067

[62] López Bastida J, Oliva J, Antoñanzas F, García-Altés A, Gisbert R, Mar J, et al. [A proposed guideline for economic evaluation of health technologies]. Gac Sanit 2009; 24: 154–170 (in Spanish). DOI: 10.1016/j.gaceta.2009.07.011 19959258

[63] NICE. NICE Health Technology Evaluations: the manual (PMG36) 2022 [accessed 2022 Jul 2]. Available from: https://www.nice.org.uk/process/pmg36

[64] Cavalli G, Cariddi A, Ferrari J, Suzzi B, Tomelleri A, Campochiaro C, et al. Living with fibromyalgia during the COVID-19 pandemic: mixed effects of prolonged lockdown on the well-being of patients. Rheumatol 2021; 60: 465–467. DOI: 10.1093/rheumatology/keaa738 PMC7717382 33188686

[65] Schweiger V, Secchettin E, Perini G, Martini A, Donadello K, Gottin L, et al. Quality of life and psychological assessment in patients with fibromyalgia syndrome during COVID-19 pandemic in Italy : prospective observational study. Signa Vitae 2022; 18: 41–46. DOI: 10.22514/sv.2021.127

[66] Colas C, Jumel A, Vericel M, Barth N. Understanding experiences of fibromyalgia patients involved in the Fimouv study during COVID-19 lockdown Front Psuchol 2021; 12: 1–9. DOI: 10.3389/fpsyg.2021.645092 PMC8329548 34354626

[67] Carrillo-de-la-Peña MT, González-Villar A, Triñanes Y. Effects of the COVID-19 pandemic on chronic pain in Spain : a scoping review. PAIN reports 2021; 6: e899. DOI: 10.1097/PR9.0000000000000899 33615089 PMC7889369

[68] Arfuch VM, Gonçalves AQ, Caballol Angelats R, Martín CA, Carrasco-Querol N, Sol MCS, et al. Patients’ appraisals about a multicomponent intervention for fibromyalgia syndrome in primary care: a focus group study. Int J Qual Stud Health Well-being 2021; 16: 2005760. DOI: 10.1080/17482631.2021.2005760 34839810 PMC8843386

[69] O’Sullivan AK, Thompson D, Drummond MF. Collection of health-economic data alongside clinical trials: is there a future for piggyback evaluations? Value Health 2005; 8: 67–79. DOI: 10.1111/j.1524-4733.2005.03065.x 15841896

