# Supplementary material for: COST-UTILITY OF A MULTICOMPONENT INTERVENTION FOR FIBROMYALGIA VERSUS USUAL CARE: A PRAGMATIC RANDOMISED CONTROLLED TRIAL
Source: J Rehabil Med. 2023 Dec 27;55:12361. doi: 10.2340/jrm.v55.12361 (PMC10753594; doi:10.2340/jrm.v55.12361)
Supplement: Supplementary file 1 [file JRM-55-12361-s1.pdf]

Supplementary material has been published as submitted. It has not been copyedited, typeset or checked for scientific content by Journal of Rehabilitation Medicine

Table SI. MCI programme implementation scheme

Basic Health Areas in Terres de L'Ebre region		Wave 1 April-June 2017	Wave 2 September 2017-February 2018	Wave 3 April-December 2018	Wave 4 April-June 2019	Wave 5 October 2019-February 2020
**PCC1**	AMPOSTA and ST BÀRBARA		x	x		
**PCC2**	DELTEBRE		x			
**PCC3**	FLIX	x			x	
**PCC4**	ALDEA and AMPOLLA	x		x	x	
**PCC5**	AMETLLA	x		x		
**PCC6**	MORA	x		x		
**PCC7**	LA RÀPITA and ALCANAR	x	x	x		x
**PCC8**	TERRA ALTA	x		x	x	
**PCC9**	TORTOSA east		x	x	x	x
**PCC10**	TORTOSA west		x	x	x	x
**PCC11**	ULLDECONA and LA SÉNIA					
	Sample included (n, %)	70 (23.6%)	53 (17.9%)	43 (14.5%)	53 (17.9%)	78 (26.3%)

MCI: multicomponent intervention; PCC: primary care centre.

Table SII. Costing matrix for direct healthcare services and indirect costs

	TYPE OF SERVICE	UNIT COST (euros)	SOURCE
**Direct Costs**	Visits at the healthcare centre		
Primary care	General practitioner, no urgent	50	Departament de salut (2020). Order SLT/71/2020, June 2. Diari Oficial de la Generalitat de Catalunya, number 8153 June 12, 2020
	Nurse, no urgent	35	
	Rehabilitation	30	
	Physiotherapist, low complexity	118	
	Basic blood tests	55	
	Emergency, without stay	105	
Specialised medical care (Hospital)	Traumatology	171/80	
	Psychiatry	171	Departament de salut (2020). Order SLT/63/2020, March 8. Diari Oficial de la Generalitat de Catalunya, number 8134 May 15, 2020.
	Rehabilitation, B level	128.36	Departament de salut (2021). Order SLT/82/2021, April 19. Diari Oficial de la Generalitat de Catalunya, number 8392, April 22, 2021.
	Emergency, no urgent and without stay	130	Departament de salut (2020). Order SLT/71/2020, June 2. Diari Oficial de la Generalitat de Catalunya, number 8153 June 12, 2020
	Other practices, external referrals	171/80	Departament de salut (2020). Order SLT/71/2020, June 2. Diari Oficial de la Generalitat de Catalunya, number 8153 June 12, 2020
	Hospital discharge	905.52	Departament de salut (2020). Order SLT/91/2020, June 17. Diari Oficial de la Generalitat de Catalunya, número 8159 June 22, 2020.
Diagnostic imaging techniques	Identified prices in the sample according to consumption	9.7 to 220*	Departament de salut (2020). Order SLT/71/2020, June 2. Diari Oficial de la Generalitat de Catalunya, number 8153 June 12, 2020 Departament de salut (2012) Order SLT/42/2012, February 24. Diari Oficial de la Generalitat de Catalunya, number 6079 March 2 2012.
Pharmacological treatment	All kind of prescribed drugs. Cost of treatment per day (CTD)	Various	Spanish Health Ministry. Specific formal consultation.
**Indirect Costs**			
Productivity loss	Absenteeism from work due to sick leave days. Cost per day based on the average labour salary for the third trimester of 2021.	91.7	Spanish National Statistics Institute (INE)
MCI programme**	Per participant per group programme	60	

CTD: Cost per treatment per day; MCI: multicomponent intervention.

* When prices were missing from the 2020 price list, data was collected from 2012. In these cases, prices were updated according to the calculation of variations in the consumer price index which registers an upward variation of 7.3% until June 2020. (Spanish National Institute of Statistics, INE https://www.ine.es/varipc/)

** The MCI cost per participant was estimated based on the actual professionals' services expenditure and the hours dedicated to the programme given a mean of 10 patients per group. Costs per professional were GP, 43,90 euros/hour; Nurse, 26,36 euros/hour; Physiotherapist, 22,50 euros/hour; Psychologist, 23,7 euros/hour.

Table SIII. Costing matrix for weighted direct primary healthcare services included in the sensitivity analysis

	TYPE OF SERVICE	UNIT COST (euros)	SOURCE
**Direct Costs**	Visits at the healthcare centre		
Primary care	General practitioner	78.64	Catalonian Primary Care Services Information System (SISAP). Requested in July 2022.
	Nurse	55.08	

Table SIV. Distribution of the sociodemographic and clinical characteristics of the non-included individuals

	**Intervention**					**Control**				
	**Included** (n = 161)	**Dropouts** (n = 24)	*p-value*	**Follow-up losses** (n = 35)	*p-value*	**Included** (n = 136)	**Dropouts** (n = 11)	*p-value*	**Follow-up losses** (n = 29)	*p-value*
**Sociodemographic characteristics**										
Sex			1		1			1		0.588
female	157 (98%)	24 (100%)		34 (97%)		131 (96%)	11 (100%)		29 (100%)	
male	4 (2.5%)	0 (0%)		1 (2.9%)		5 (3.7%)	0 (0%)		0 (0%)	
Age	58 (50, 65)	61 (57, 66)	0.077	60 (53, 66)	0.462	59 (52, 68)	51 (44, 62)	0.085	56 (51, 64)	0.473
Birth country			0***		0***			0.117		0***
Spain	159 (99%)	18 (75%)		25 (71%)		131 (96%)	10 (91%)		19 (66%)	
Other	2 (1.2%)	1 (4.2%)		0 (0%)		5 (3.7%)	0 (0%)		1 (3.4%)	
Missing	0 (0%)	5 (21%)		10 (29%)		0 (0%)	1 (9.1%)		9 (31%)	
Education			0.06		0.744			0.559		0.292
None	36 (22%)	8 (33%)		9 (26%)		16 (12%)	1 (9.1%)		7 (24%)	
Primary	71 (44%)	9 (38%)		13 (37%)		66 (49%)	8 (73%)		12 (41%)	
Secondary	34 (21%)	1 (4.2%)		7 (20%)		29 (21%)	1 (9.1%)		6 (21%)	
Tertiary	4 (2.5%)	0 (0%)		2 (5.7%)		16 (12%)	0 (0%)		1 (3.4%)	
Missing	16 (9.9%)	6 (25%)		4 (11%)		9 (6.6%)	1 (9.1%)		3 (10%)	
Marital status			0.665		0.952			0.456		0.663
Married	119 (74%)	17 (71%)		25 (71%)		89 (65%)	9 (82%)		23 (79%)	
Divorced	16 (9.9%)	2 (8.3%)		5 (14%)		19 (14%)	0 (0%)		2 (6.9%)	
Single	6 (3.7%)	0 (0%)		1 (2.9%)		7 (5.1%)	1 (9.1%)		0 (0%)	
Widow/er	5 (3.1%)	1 (4.2%)		1 (2.9%)		12 (8.8%)	0 (0%)		2 (6.9%)	
Missing	15 (9.3%)	4 (17%)		3 (8.6%)		9 (6.6%)	1 (9.1%)		2 (6.9%)	
Living alone	6 (3.7%)	1 (4.2%)	1	2 (5.7%)	0.635	19 (14%)	0 (0%)	0.36	0 (0%)	0.027*

Living with partner	117 (73%)	11 (46%)	0.008**	23 (66%)	0.409	92 (68%)	8 (73%)	1	21 (72%)	0.616
Living with partner & children	37 (23%)	5 (21%)	0.815	6 (17%)	0.449	34 (25%)	3 (27%)	1	4 (14%)	0.193
Living with partner, children & parents	2 (1.2%)	0 (0%)	1	0 (0%)	1	1 (0.7%)	0 (0%)	1	0 (0%)	1
Living with others	4 (2.5%)	2 (8.3%)	0.175	4 (11%)	0.035*	7 (5.1%)	1 (9.1%)	0.472	2 (6.9%)	0.659
Working condition			0.157		0.054			0.029*		0.785
Employed	59 (37%)	5 (21%)		15 (43%)		46 (34%)	3 (27%)		9 (31%)	
Unemployed	13 (8.1%)	1 (4.2%)		3 (8.6%)		16 (12%)	2 (18%)		3 (10%)	
Retired	32 (20%)	3 (13%)		6 (17%)		38 (28%)	0 (0%)		6 (21%)	
Disabled	16 (9.9%)	3 (13%)		7 (20%)		7 (5.1%)	3 (27%)		3 (10%)	
Homemaker	26 (16%)	6 (25%)		0 (0%)		20 (15%)	2 (18%)		6 (21%)	
Missing	15 (9.3%)	6 (25%)		4 (11%)		9 (6.6%)	1 (9.1%)		2 (6.9%)	
Occupational class			0.117		0.797			0.41		0.347
I: Professionals	7 (4.3%)	0 (0%)		2 (5.7%)		16 (12%)	0 (0%)		0 (0%)	
II: Intermediate occupations	12 (7.5%)	0 (0%)		2 (5.7%)		9 (6.6%)	1 (9.1%)		2 (6.9%)	
III: Skilled non-manual workers	21 (13%)	3 (13%)		6 (17%)		15 (11%)	1 (9.1%)		3 (10%)	
IVa: Skilled manual workers	21 (13%)	2 (8.3%)		6 (17%)		12 (8.8%)	0 (0%)		3 (10%)	
IVb: Other manual workers	82 (51%)	11 (46%)		14 (40%)		71 (52%)	6 (55%)		16 (55%)	
Missing	18 (11%)	8 (33%)		5 (14%)		13 (9.6%)	3 (27%)		5 (17%)	
**Clinical characteristics**										
Years since FMS diagnosis	6.0 (1.0, 11.0)	9.0 (4.5, 12.0)	0.252	7.0 (2.0, 11.0)	0.682	6.0 (2.0, 11.0)	7.0 (2.0, 12.0)	0.965	8.0 (2.0, 11.0)	0.605
Having a family history of FMS	46 (29%)	8 (33%)	0.632	11 (31%)	0.736	38 (28%)	3 (27%)	1	5 (17%)	0.233
Physical trigger factor	32 (20%)	5 (21%)	1	5 (14%)	0.444	26 (19%)	1 (9.1%)	0.69	1 (3.4%)	0.05*
Psychological trigger factor	43 (27%)	7 (29%)	0.8	7 (20%)	0.409	40 (29%)	2 (18%)	0.729	7 (24%)	0.568
Physical activity as trigger factor	37 (23%)	4 (17%)	0.487	5 (14%)	0.256	38 (28%)	2 (18%)	0.728	6 (21%)	0.423
Stress as trigger factor	75 (47%)	7 (29%)	0.109	12 (34%)	0.184	65 (48%)	3 (27%)	0.189	3 (10%)	0***
Total symptoms	6.0 (4.0, 8.0)	4.0 (2.0, 7.3)	0.039*	6.00 (4.0, 8.0)	0.878	7.00 (4.0, 8.0)	4.00 (3.5, 7.0)	0.21	4.00 (2.0, 5.0)	0***
HADS scale			0.762		0.955			0.008**		0.813
(0,14]	22 (14%)	4 (17%)		4 (11%)		29 (21%)	0 (0%)		5 (17%)	
(14,22]	54 (34%)	6 (25%)		13 (37%)		48 (35%)	1 (9.1%)		9 (31%)	
(22,42]	83 (52%)	14 (58%)		18 (51%)		58 (43%)	9 (82%)		15 (52%)	
Missing	2 (1.2%)	0 (0%)		0 (0%)		1 (0.7%)	1 (9.1%)		0 (0%)	

FIQR total score	68 (53, 81)	72 (67, 83)	0.134	70 (46, 80)	0.966	69 (56, 82)	71 (50, 84)	0.828	68 (47, 83)	0.535
Missing	1	0		0		0	0		0	
Presence of comorbidities	90 (56%)	11 (46%)	0.355	24 (69%)	0.168	93 (68%)	9 (82%)	0.504	12 (41%)	0.006**

HADS: Hospital Anxiety and Depression Scale; FIQR: Revised Fibromyalgia Impact Questionnaire
Note:
(i) Numerical continuous variables are presented with a Median (IQR); Categorical variables are presented with n (%)
(ii) P-values were estimated to explore statistical differences between non-included cases and the included ones by using Fisher's exact test, Wilcoxon rank sum test, or Pearson's Chi-squared test

*sig.≤0,05 **sig.≤0,01 ***sig.≤0,001

Table SV. List of explored comorbidities at baseline

**Diagnostic**	CI-10 code
Other mental disorders due to known physiological conditions	F06
Persistent mood [affective] disorders	F34
Other anxiety disorders	F41
Anxiety disorder, unspecified	F41.9
Specific personality disorders	F60
Borderline personality disorder	F60.3
Bipolar disorder	F31
Osteoporosis without current pathological fracture	M81
Other specified disorders of bone density and structure	M85.8
Other anaemias	D64
Type 2 diabetes mellitus	E11
Lupus erythematosus	L93
Vitamin D deficiency, unspecified	E55.9
Other and unspecified osteoarthritis	M19
Rheumatoid arthritis, unspecified	M06.9
Unspecified osteoarthritis, unspecified site	M19.90
Polymyalgia rheumatica	M35.3
Myopia	H52.1
Chronic fatigue, unspecified	R53.82
Other types of fatigue	R53.83

Other cervical displacement	M50.2
Pain and other conditions associated with female genital organs and menstrual cycle	N94
Absent, scanty and rare menstruation	N91
Endometriosis	N80
Noninflammatory disorders of the ovary, fallopian tube and broad ligament	N83
Pelvic and perineal pain	R10.2
